# Retraction: Early detection of plant leaf diseases using stacking hybrid learning

**DOI:** 10.1371/journal.pone.0328535

**Published:** 2025-07-21

**Authors:** The PLOS One Editors

The *PLOS One* Editors retract this article [1] due to concerns about:

- Compliance with PLOS policies on Artificial Intelligence Tools and Technologies.
- The use of terminology that differs from standards in the field (e.g., neural community instead of neural network).
- Adherence to the journal’s requirements regarding methodology reporting.
- The reliability of the reported results and conclusions.

The author did not agree with the retraction.
